# Neuroinflammation and White Matter Alterations in Obesity Assessed by Diffusion Basis Spectrum Imaging

**DOI:** 10.3389/fnhum.2019.00464

**Published:** 2020-01-14

**Authors:** Amjad Samara, Tatianna Murphy, Jeremy Strain, Jerrel Rutlin, Peng Sun, Olga Neyman, Nitya Sreevalsan, Joshua S. Shimony, Beau M. Ances, Sheng-Kwei Song, Tamara Hershey, Sarah A. Eisenstein

**Affiliations:** ^1^Department of Psychiatry, Washington University School of Medicine, St. Louis, MO, United States; ^2^Department of Neurology, Washington University School of Medicine, St. Louis, MO, United States; ^3^Mallinckrodt Institute of Radiology, Washington University School of Medicine, St. Louis, MO, United States; ^4^Department of Psychological & Brain Sciences, Washington University School of Medicine, St. Louis, MO, United States

**Keywords:** obesity, white matter, neuroinflammation, diffusion tensor imaging, diffusion basis spectrum imaging

## Abstract

Human obesity is associated with low-grade chronic systemic inflammation, alterations in brain structure and function, and cognitive impairment. Rodent models of obesity show that high-calorie diets cause brain inflammation (neuroinflammation) in multiple regions, including the hippocampus, and impairments in hippocampal-dependent memory tasks. To determine if similar effects exist in humans with obesity, we applied Diffusion Basis Spectrum Imaging (DBSI) to evaluate neuroinflammation and axonal integrity. We examined diffusion-weighted magnetic resonance imaging (MRI) data in two independent cohorts of obese and non-obese individuals (Cohort 1: 25 obese/21 non-obese; Cohort 2: 18 obese/41 non-obese). We applied Tract-based Spatial Statistics (TBSS) to allow whole-brain white matter (WM) analyses and compare DBSI-derived isotropic and anisotropic diffusion measures between the obese and non-obese groups. In both cohorts, the obese group had significantly greater DBSI-derived restricted fraction (DBSI-RF; an indicator of neuroinflammation-related cellularity), and significantly lower DBSI-derived fiber fraction (DBSI-FF; an indicator of apparent axonal density) in several WM tracts (all corrected *p* < 0.05). Moreover, using region of interest analyses, average DBSI-RF and DBSI-FF values in the hippocampus were significantly greater and lower, respectively, in obese relative to non-obese individuals (Cohort 1: *p* = 0.045; Cohort 2: *p* = 0.008). Hippocampal DBSI-FF and DBSI-RF and amygdalar DBSI-FF metrics related to cognitive performance in Cohort 2. In conclusion, these findings suggest that greater neuroinflammation-related cellularity and lower apparent axonal density are associated with human obesity and cognitive performance. Future studies are warranted to determine a potential role for neuroinflammation in obesity-related cognitive impairment.

## Introduction

Obesity is a rapidly growing epidemic around the world. According to the World Health Organization, in 2016 more than 1.9 billion adults were overweight and 650 million (about 9% of the world population) were obese (≥30 kg/m^2^) (WHO, 2018). Obesity is associated with comorbidities including type 2 diabetes, hypertension, heart disease, and cancer (Haslam and James, 2005). In addition, obesity is linked to cognitive deficits and is a risk factor for Alzheimer’s disease (Miller and Spencer, 2014; Walker and Harrison, 2015; Alford et al., 2018). These latter features have raised the question of how obesity and its comorbidities may influence brain function and structure. Neuroimaging studies have found both structural and functional abnormalities in obesity, but the mechanisms underlying these differences are not well understood (Devoto et al., 2018; van Galen et al., 2018; Garcia-Garcia et al., 2019). One potential mechanism for brain structural and functional findings is brain inflammation (neuroinflammation), but this has not been explored thoroughly in humans (Guillemot-Legris and Muccioli, 2017).

Obesity is a disease of low-grade chronic systemic inflammation that affects many body organs (Gregor and Hotamisligil, 2011). Also, evidence from rodent models shows that obesity causes neuroinflammation (Guillemot-Legris et al., 2016). Similarly, in humans with obesity, postmortem brain examination shows evidence of gliosis and abnormal microglia activation in the hypothalamus and altered mRNA expression of inflammatory markers in frontal cortex suggestive of neuroinflammation (Baufeld et al., 2016; Lauridsen et al., 2017). Hypercaloric diet induces breakdown of the BBB, allowing pro-inflammatory cytokines to enter the CNS (Guillemot-Legris et al., 2016; Stranahan et al., 2016; Guillemot-Legris and Muccioli, 2017) and promotes peripheral macrophage infiltration to the brain (Stranahan et al., 2016), which subsequently contributes, among other factors such as increased peripheral free fatty acid circulation (O’Brien et al., 2017), to obesity-associated neuroinflammation. Intriguingly, hippocampal neuroinflammation causes deficits in memory tasks in rodent models of obesity (Pistell et al., 2010; Beilharz et al., 2016; Cope et al., 2018). In humans, higher adiposity is generally associated with poorer cognitive performance in a variety of measures, yet the underlying mechanism is not entirely understood (Wright et al., 2016; Gameiro et al., 2017; Tsai et al., 2017). Taken together, it is reasonable to hypothesize that obesity-related neuroinflammation impacts the function and structure of the human brain and could be an underlying mechanism of obesity-associated cognitive impairment.

Evaluation of obesity-associated neuroinflammation in humans via imaging is technically challenging and there are few research studies in this area. Measuring specific processes related to neuroinflammation (e.g., microglial activation) with neuroimaging is possible via PET with radiotracers (e.g., TSPO radiotracer) (Vivash and O’Brien, 2016; Alam et al., 2017). However, these PET radiotracers vary in specificity, and some individuals (∼34% of Caucasians) have genotypes that confer very low to mixed binding affinity for TSPO ligands (Owen et al., 2012). Other research groups utilized MRI-based techniques to evaluate obesity-associated neuroinflammation. For example, alterations in T2-weighted MRI signal intensity (an indicator of gliosis) in the hypothalamus have been found in obese individuals (Thaler et al., 2012; Schur et al., 2015; Kreutzer et al., 2017). Also, plasma fibrinogen, a driver of inflammation, has been related to alterations in diffusivity characteristics of extra-hypothalamic brain regions including orbitofrontal cortex and amygdala in overweight and obese individuals (Cazettes et al., 2011). Interestingly, a recent study has also shown sex-specific effects of central adiposity and systemic inflammatory markers on limbic system microstructure (Metzler-Baddeley et al., 2019).

At the same time, a large number of neuroimaging studies have focused on the impact of obesity on WM microstructure using standard DTI modeling (Kullmann et al., 2015; Alfaro et al., 2018). DTI models a single diffusion tensor within an image voxel, to derive the standard diffusion tensor metrics (AD, RD, FA). Using this standard model, several studies have found that individuals with higher BMI have lower FA in many WM tracts (Marks et al., 2011; Mueller et al., 2011; Stanek et al., 2011; Verstynen et al., 2012; Karlsson et al., 2013; Xu et al., 2013; Lou et al., 2014; Bolzenius et al., 2015; He et al., 2015; Kullmann et al., 2015; Kullmann et al., 2016; Mazza et al., 2017; Papageorgiou et al., 2017; Alfaro et al., 2018) and mixed effects on AD and RD (Mueller et al., 2011; Xu et al., 2013; Kullmann et al., 2016; Mazza et al., 2017; Papageorgiou et al., 2017). In the healthy brain or disease conditions with limited edema and inflammation, lower FA and AD reflects impaired overall WM integrity and axonal injury, respectively, while greater RD reflects myelin damage (Wheeler-Kingshott and Cercignani, 2009; Winklewski et al., 2018). However, neuroinflammation-related processes such as cellularity and edema may confound standard DTI modeling, lead to mixed effects on AD and RD, and decrease the sensitivity and specificity to detect WM microstructural alterations (Winklewski et al., 2018).

In recent years, a novel data-driven DBSI (Wang et al., 2011; Wang et al., 2014) approach has been developed that shows sensitivity to both neuroinflammation and WM microstructural alterations. DBSI resolves intra-voxel partial volume effects arising from anisotropic and isotropic diffusion signals, and models both simultaneously to obtain the best estimation of anisotropic and isotropic diffusion tensors. Anisotropic tensor components modeled by DBSI consider water diffusion of WM tracts within the image voxel, deriving the rate of water diffusion parallel to the axon (DBSI-axial diffusivity or DBSI-AD) and perpendicular to the axon (DBSI-radial diffusivity or DBSI-RD) or fiber-tract specific diffusion anisotropy (DBSI-fractional anisotropy or DBSI-FA) reflecting the integrity of axon bundles. DBSI-derived fiber fraction (DBSI-FF) indicates axonal density. Simultaneously, DBSI models restricted isotropic diffusion into DBSI-restricted fraction (DBSI-RF; an indicator of resident and neuroinflammation-related cellularity) and non-restricted diffusion into DBSI-hindered fraction (DBSI-HF; an indicator of tissue edema). DBSI-derived isotropic measures (DBSI-RF and DBSI-HF) are sensitive to inflammation-related cellularity and tissue edema, respectively (Cross and Song, 2017), and both are present in neuroinflammation (Frohman et al., 2006; Stamatovic et al., 2006). Validation studies of DBSI in animal models have shown that this method can differentiate axonal injury, demyelination, and neuroinflammation in white and gray matter (Wang et al., 2014; Cross and Song, 2017; Zhan et al., 2018). In humans, DBSI has been used to detect indicators of neuroinflammation in MS (Chiang et al., 2014), cervical spondylotic myelopathy (Murphy et al., 2016), traumatic spinal cord injury (Sun et al., 2017), HIV (Strain et al., 2017), and Alzheimer’s disease (Wang et al., 2019). Importantly, when neuroinflammation is present, DBSI can provide further insight into WM microstructural integrity (Wang et al., 2011; Chiang et al., 2014; Wang et al., 2014; Wang et al., 2015; Murphy et al., 2016; Cross and Song, 2017; Lin et al., 2017; Strain et al., 2017; Sun et al., 2017; Shirani et al., 2018; Zhan et al., 2018; Lin et al., 2019).

The goal of the current study was to apply DBSI in humans to evaluate the presence of neuroinflammation and provide further insight into WM microstructural integrity in obesity. DBSI-derived metrics may also help resolve some of the conflicting findings from the DTI literature in obesity (Kullmann et al., 2015). We hypothesized that obese individuals would have greater DBSI-RF (an indicator of increased neuroinflammation-related cellularity), greater DBSI-HF (an indicator of increased edema), and lower DBSI-FF (an indicator of decreased apparent axonal density) compared to non-obese individuals. We tested these hypotheses in a cohort of obese and non-obese individuals recruited specifically for a study of brain alterations in obesity (Cohort 1). We then examined a more heterogeneous convenience sample to confirm the presence of similar patterns related to BMI status (Cohort 2). Since obese individuals show impaired cognitive function relative to non-obese individuals (Wright et al., 2016; Gameiro et al., 2017; Tsai et al., 2017), hippocampal neuroinflammation causes impairment on memory tasks in rodent models of obesity (Pistell et al., 2010; Beilharz et al., 2016; Cope et al., 2018), and the hippocampus and amygdala operate together to form emotion-associated memory (Yang and Wang, 2017), we selected the hippocampus and amygdala to perform region of interest (ROI) analyses and explored the presence of similar alterations in these regions and their relation to cognitive performance.

## Materials and Methods

### Participants

In both cohorts, obesity was defined as ≥30 kg/m^2^. Non-obesity was defined as ≤25 kg/m^2^. All studies were approved by the Washington University School of Medicine Human Research Protection Office and were carried out in accordance with the principles expressed in the Declaration of Helsinki. All participants gave written, informed consent prior to participation.

Cohort 1: Healthy obese and non-obese adults were recruited through an online research participant database at Washington University, advertisements, and word of mouth for a neuroimaging study on obesity. All participants were assessed for the presence of diabetes with an oral glucose tolerance test and excluded from further participation if glucose or hemoglobin A1c levels met American Diabetes Association criteria for Type 2 diabetes (American Diabetes Association, 2016). Participants were also assessed with a detailed history, including neurological and physical examinations, psychiatric interviews using the Structured Interview for DSM-IV-TR Axis I Disorders (SCID) (First et al., 2002), and routine blood tests. Volunteers were excluded for history of medical problems as well as other significant neurological, cerebrovascular, cardiovascular, or psychiatric diagnosis (DSM-IV Axis I disorders except for specific phobias), head trauma, any current or recent dopaminergic drug use (e.g., stimulants, agonists, bupropion, neuroleptics or metoclopramide), current heavy alcohol use (males > 2 drinks per day, females > 1 drink per day) or illicit drug use, history of substance abuse or dependence, and IQ < 80 as measured by the Wechsler Abbreviated Scale of Intelligence (WASI) (Wechsler, 1999). Data from individuals in this sample have been reported previously (Eisenstein et al., 2013; Eisenstein et al., 2015a, b; Pepino et al., 2016).

Cohort 2: Healthy obese and non-obese adults were recruited through an online research database at Washington University and flyers to be a control group for ongoing studies. Exclusion criteria included self-reported diabetic medication use or unknown diabetic medication status, current or past history of confounding neurological disorders, depression as assessed by the Beck Depression Inventory II (BDI-II) (Beck et al., 1996), current alcohol or substance abuse, head injury with loss of consciousness greater than 30 min, claustrophobia or seizures, and fewer than 8 years of education. Data from some individuals in this sample have been reported previously (Strain et al., 2017).

### BMI Measures

Body mass index was calculated as kg/m^2^ in both cohorts. Cohort 1: Height and weight measurements were taken by a trained nurse. Cohort 2: Height and weight were self-reported by participants.

#### Neuropsychological Performance

As described previously (Strain et al., 2017), individuals in Cohort 2 completed a cognitive test battery that included executive function, verbal and visuospatial learning and memory, and psychomotor speed. These included the Wechsler Adult Intelligence Scale III [WAIS-III including digit span, digit symbol, symbol search, and letter number sequencing subtests (Wechsler, 1997)]; Trail-making Test Parts A and B (Reitan, 1958); Multilingual Aphasia Examination verbal fluency subtest (Benton and Hamsher, 1976); F-A-S test (Spreen and Benton, 1977); animal (category) fluency (Goodglass and Kaplan, 1972); Delis-Kaplan Executive Function System [D-KEFS including Color-Word Interference Task (Delis et al., 2001)]; Hopkins Verbal Learning Test (HVLT) learning and recall (Brandt, 1991); Brief Visuospatial Memory Test-Revised (BVMT-R) (Benedict, 1997); Grooved Peg Board (Baser and Ruff, 1987); and finger-tapping test (FTT) (Schmitt, 2013). The Wide Range Achievement Test 3 (WRAT3) (Snelbaker et al., 2001) was also administered.

### MRI Acquisition

Cohort 1: Magnetic resonance imaging scanning was performed on a Siemens Trio 3T scanner with a 20 channel head coil. Structural magnetic resonance T1-weighted anatomical images were obtained using a 3-D MPRAGE sequence [sagittal orientation, repetition time (TR) = 2400 ms, echo time (TE) = 3.16 ms, inversion time (TI) = 1000 ms, voxel resolution = 1 × 1 × 1 mm^3^, frames = 176, flip angle = 8°, FOV = 256 × 256 mm]. We acquired two echo planar DTI sequences, of similar phase encoding direction, with 27 volumes each (transverse orientation, 2 × 2 × 2 mm^3^ voxels, TR = 12,300 ms, TE = 108 ms, flip angle = 90°, 25 directions, *b*-values ranging from 0 to 1400 s/mm^2,^ and two non-diffusion weighted images).

Cohort 2: Magnetic resonance imaging scanning was performed on the same Siemens Trio 3T scanner with a 12 channel head coil. Structural magnetic resonance T1-weighted anatomical images were obtained using the 3-D MPRAGE sequence described for Cohort 1. Two sequential diffusion-weighted scans, of similar phase encoding direction, were obtained (transverse orientation, 2 × 2 × 2 mm^3^ voxels, TR = 9,900 ms, TE = 102 ms, flip angle = 90°, 23 directions, *b*-values ranging from 0 to 1400 s/mm^2^, and one non-diffusion weighted image).

### Image Preprocessing and DTI Processing

For both cohorts, all DTI volumes were manually inspected to exclude the presence of large artifacts. FMRIB Software Library (FSL) (Smith et al., 2004) was used to perform all preprocessing steps and fit the DTI diffusion tensor model at each imaging voxel. Non-brain tissue was removed using FSL BET (brain extraction tool) (Smith, 2002), followed by motion and eddy-current distortions correction. Field maps were not acquired as part of these studies and thus corrections for susceptibility-induced distortions were not performed. For DTI analyses, FSL DTIFIT tool was used to compute diffusivities from fitting the diffusion tensor model and to generate DTI-FA (DTI-fractional anisotropy), DTI-MD (DTI-mean diffusivity), DTI-RD (DTI-radial diffusivity), and DTI-AD (DTI-axial diffusivity) volumes for each subject. DTI-derived image volumes for each participant were subsequently processed through the TBSS (Smith et al., 2006) pipeline to allow for whole-brain WM voxel-wise analyses as described below.

Since head motion during MRI scans is positively related to and shares genetic factors with BMI (Hodgson et al., 2017), and because registration-based correction methods do not exclude the effects of head motion entirely, we also computed motion parameters as described by Yendiki et al. (2014). These motion parameters include average volume-by-volume translation, average volume-by-volume rotation, percentage of slices with signal drop-out, and signal drop-out severity. In order to obtain these motion measures, we completed the image correction and quality assessment steps of the TRACULA pipeline (TRActs Constrained by UnderLying Anatomy), without running the WM pathways reconstruction steps (Yendiki et al., 2011). TRACULA-derived average volume-by-volume translation and average volume-by-volume rotation were included as regressors in subsequent voxel-wise and statistical analyses. The readout of percentage of slices with signal drop-out and signal drop-out severity were 0 and 1, respectively, for every participant in both cohorts.

### DBSI Processing

Diffusion basis spectrum imaging measures were calculated using in-house software scripted in MATLAB and Statistics Toolbox Release (2012), and as first described in Wang et al. (2011). Unlike conventional DTI modeling, DBSI modeling simultaneously differentiates and quantifies several intravoxel pathological processes (axonal injury/loss, axonal demyelination, neuroinflammation-related cellularity, and vasogenic edema) by assigning a dedicated diffusion tensor for each of these pathological processes. While DTI-derived FA quantifies the degree of anisotropy for the whole image voxel, DBSI estimates anisotropy of fiber tracts within the image voxel without being confounded by isotropic diffusion. The total diffusion signal (*S*_*k*_) measured by DBSI includes both anisotropic (*A*_*k*_) and isotropic (*I*_*k*_) diffusion tensor components, and the weighted sum of these components is presented in Eq. 1.

(1) $$S_{k}=\sum_{i=1}^{N_{A\,n\,i\,s\,o}}f_{i}\,e^{-\left|\vec{b_{k}}\right|.\lambda_{{⊥}_{i}}}\,e^{-\left|\vec{b_{k}}\right|.\left(\lambda_{{∥}_{i}}-\lambda_{{⊥}_{i}}\right).\cos^{2}\Phi_{i\,k}}+\int_{a}^{b}f\,\left(D\right)\,e^{-\left|\vec{b_{k}}\right|\,D}\,dD\left(k=1,2,3,\ldots ,k\right).$$

Where *S*_*k*_ and $\vec{b_{k}}$ are the signal and *b*-value of the *k*^*th*^ diffusion gradient; *N*_*Aniso*_ is the number of anisotropic tensors, Φ_*ik*_ is the angle between the principal direction of the *i*^th^ anisotropic tensor and the *k*^th^ diffusion gradient; λ_||*i*_ and λ_⊥*i*_ are the AD and RD of the *i*^th^ anisotropic tensor, *f*_*i*_ is the signal intensity fraction for the *i*^th^ anisotropic tensor, and *a* and *b* are the isotropic diffusion spectrum *f*(*D*) low and high diffusivity limits.

Moreover, DBSI assesses isotropic diffusion tensor signal distribution within the whole spectrum of apparent isotropic diffusivity (resulting from intracellular and sub-cellular structures, and edematous extracellular tissue). Through previous experimental analyses (Wang et al., 2011; Wang et al., 2015), we grossly grouped isotropic diffusion as restricted diffusion (*D* ≤ 0.3 μm^2^/ms; a proxy measure of water diffusion in the intracellular compartment hence cellularity), and non-restricted isotropic diffusion (*D* > 0.3 μm^2^/ms; a proxy measure of water diffusion in the extracellular space). By solving the DBSI model, we obtain a group of anisotropic and isotropic metrics that include: DBSI-FA (indicates overall WM integrity), DBSI-AD (indicates axonal loss/injury), DBSI-RD (indicates myelin loss), DBSI-fiber fraction or DBSI-FF (indicates apparent axonal density), DBSI-RF (*D* ≤ 0.3 μm^2^/ms; indicates inflammation-related cellularity), and DBSI-hindered fraction or DBSI-HF (*D* > 0.3 μm^2^/ms; indicates extracellular tissue edema). DBSI-derived image volumes for each subject were subsequently processed through the TBSS pipeline to allow for whole-brain WM voxel-wise analyses as described below.

### TBSS and Voxel-Wise Analyses

Post-processing and voxel-wise analyses of DTI- and DBSI-derived metrics were completed with TBSS (Smith et al., 2006). DTI-FA images were used to create an average WM skeleton. First, all DTI-FA were slightly eroded and end slices were excluded to remove potential outliers from diffusion tensor fitting. Secondly, all images were non-linearly registered to FMRIB58-FA standard-space image as a target image. Aligned FA images were then averaged to create a mean FA image, and fed into the skeletonization step to create a WM skeleton using a threshold of FA > 0.2. Using the same transformation process, all DTI- and DBSI-derived images, for each subject, were projected onto the mean FA skeleton, which represents the center of WM tracts common to all subjects, and used to perform further voxel-wise and ROI analyses.

Finally, the FSL Randomize tool (Winkler et al., 2014) was applied to perform separate voxel-wise statistical analyses within each cohort, and determined which skeleton voxels were significantly different between obese and non-obese groups (*p* < 0.05, corrected for multiple comparisons). We used GLMs controlling for age, sex, and race. Also, to account for the effects of head motion, we also controlled for TRACULA-derived motion measures (volume-by-volume translation and rotation). The TFCE option was used in TBSS analysis to correct for family-wise error (Nichols and Holmes, 2002).

### Hippocampal and Amygdalar ROI and WM Tracts Analyses

In both cohorts, total hippocampal and amygdalar volumes for each individual were computed using FreeSurfer 6.0 segmentation^1^ of corresponding structural MRI images. Hippocampal and amygdalar volumes were corrected for total intracranial volumes (ICV) and compared between obese and non-obese groups. For all individuals, average DBSI-derived metrics (both anisotropic and isotropic) in the right and left hippocampus and amygdala were extracted using ROIs from the 50% thresholded Harvard-Oxford Subcortical Structural Atlas provided by the Harvard Center for Morphometric Analysis in FSL (Smith et al., 2004). Average hippocampal and amygdalar DBSI-metrics were compared between obese and non-obese groups in each cohort separately. Additionally, the JHU-ICBM-DTI-81 WM labels atlas was used to create masks to define WM ROIs for further analyses (Mori et al., 2008). To assess whether differences in DBSI metrics between obese and non-obese groups were spatially and qualitatively similar across cohorts, we computed the percentage of overlap between cohorts in all 48 WM tracts for significant differences in DBSI-RF, DBSI-FF, and DBSI-AD. Importantly, the hypothalamus was not included in our ROI analyses. In our experience, anatomical boundaries of the hypothalamus are not clearly visible on MRIs, making it difficult to be certain whether measures are not affected by partial volume effects. Therefore, we did not include this region in our analyses.

### Statistical Analyses

Differences in demographic, motion parameters, and hippocampal volume variables between non-obese and obese individuals were assessed with between-subjects Student’s *t*-tests or, if data were not normally distributed, Mann–Whitney *U* tests. Differences in race and sex distributions between obese and non-obese groups were assessed with Chi-square tests. Voxel-wise analyses compared DTI- and DBSI-derived metrics between obese and non-obese groups within each cohort separately, using GLM controlling for age, sex, race, and TRACULA-derived motion parameters (volume-by-volume translation and rotation). Further voxelwise GLM analyses determined whether BMI related to DBSI metrics of interest within each group in both cohorts. For each GLM, the FSL statistical package Randomize (Winkler et al., 2014) was used to correct for multiple comparisons via a TFCE approach with a family-wise error rate derived from 5000 Monte Carlo permutations (Nichols and Holmes, 2002). Statistical significance was thresholded at corrected *p* ≤ 0.05. Average hippocampal and amygdalar DTI- and DBSI-derived metrics were compared between obese and non-obese groups, within each cohort separately, using a multiple linear regression model with age, sex, race, average hippocampal or amgydalar volume, and motion parameters as covariates. Additionally, in Cohort 2, we used partial Pearson *r* correlations controlling for age to relate main DBSI outcomes in the hippocampus and amygdala with performance on cognitive tasks. It was not expected that these exploratory correlational analyses would survive multiple comparison correction [0.05/(19 tests × 2 brain regions) = 0.0013]. Differences in cognitive performance between obese and non-obese individuals in Cohort 2 were assessed with two-tailed between-subjects Student’s *t*-tests.

## Results

### Participants

Participant demographics and descriptive statistics for Cohort 1 and Cohort 2 are shown in Table 1.

**TABLE 1:** Demographic data and TRACULA-derived motion parameters for obese and non-obese participants in Cohort 1 and Cohort 2.

**Cohort 1**	**Non-obese**	**Obese**	***p*-value**
	**(*n* = 21)**	**(*n* = 25)**	
Age (years) mean (S.D.)	28 (5.2)	31.6 (6.4)	0.05^∗^
Sex (male/female)	5/16	4/21	0.51
Race	18 C/2 AA/1 H	13 C/12 AA	0.01^∗∗^
Body mass index (kg/m^2^) mean (S.D.)	22 (2.2)	40 (4.9)	< 0.001^∗∗∗^
Education level (years) mean (S.D.)	15.8 (1.49)	15.1 (1.82)	0.23
Volume-by-volume translation (mm) mean (S.D.)	0.96 (0.2)	1.1 (0.1)	0.03^∗^
Volume-by-volume rotation (mm) mean (S.D.)	0.0039 (0.0007)	0.0043 (0.0007)	0.08

**Cohort 2**	**Non-obese**	**Obese**	***p*-value**
	**(*n* = 41)**	**(*n* = 18)**	

Age (years) mean (S.D.)	29.5 (14.4)	29.8 (12.9)	0.12
Sex (male/female)	25/16	3/15	0.002^∗∗^
Race	23 C/16 AA/1	5 C/13 AA	0.03^∗^
	AS/1 BI		
Body mass index (kg/m^2^) mean (S.D.)	21.7 (1.7)	35.7 (4.3)	< 0.001^∗∗∗^
Education level (years) mean (S.D.)	13.2 (2.08)	13.3 (1.33)	0.55
Volume-by-volume translation (mm) mean (S.D.)	1.06 (0.1)	1.13 (0.13)	0.04^∗^
Volume-by-volume rotation (mm) mean (S.D.)	0.0042 (0.001)	0.0048 (0.001)	0.09

*^∗^, ^∗∗^, ^∗∗∗^, *p* ≤ 0.05, 0.01, 0.001 relative to non-obese. Independent Student’s *t*-test, Mann–Whitney *U* test, or Pearson Chi-Square test were used as appropriate. C, Caucasian; AA, African American; H, Hispanic; AS, Asian; BI, Bi-racial. S.D., standard deviation.*

Cohort 1: Twenty-five obese (BMI = 33.4–51 kg/m^2^) and twenty-one non-obese (BMI = 18.6–25.9 kg/m^2^) participants contributed DTI scans for analyses. Data from two individuals whose BMIs were 25.1 and 25.9 kg/m^2^ were included as non-obese since they met criteria for normal percent body fat and other metabolic parameters. Obese participants were older than non-obese participants and had a larger proportion of African Americans compared to the non-obese group. Non-obese and obese groups did not differ in sex distribution or years of education.

Cohort 2: Eighteen obese (BMI = 30–43 kg/m^2^) and forty-one non-obese (BMI = 18.5–25 kg/m^2^) participants contributed DTI scans for analyses. Sex and race distributions differed between obese and non-obese groups such that there were higher proportions of females and African Americans in the obese group. Obese and non-obese groups did not differ in age or years of education.

### Motion Parameters

TRACULA-derived motion parameters for Cohort 1 and Cohort 2 are shown in Table 1. In both cohorts, the obese group required greater volume-by-volume translation for motion correction during the DTI scans. However, neither this measure nor volume-by-volume rotation related to BMI within non-obese (*r* ≤ 0.31, *p* ≥ 0.18) or obese (*r* ≤ 0.21, *p* ≥ 0.40) groups in either cohort.

### Voxel-Wise Comparison of DBSI and DTI Metrics

**FIGURE 1:**
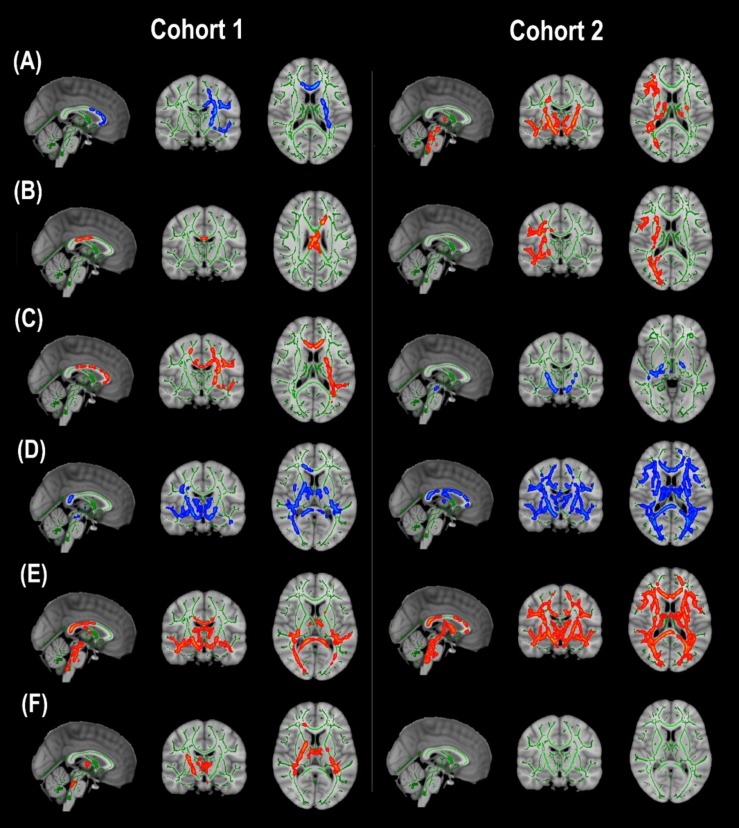
Diffusion basis spectrum imaging-derived measures of white matter integrity and indicators of neuroinflammation in Cohort 1 and Cohort 2. **(A)** DBSI-derived fractional anisotropy. **(B)** DBSI-derived axial diffusivity. **(C)** DBSI-derived radial diffusivity. **(D)** DBSI-derived fiber fraction. **(E)** DBSI-derived restricted fraction. **(F)** DBSI-derived hindered fraction. *Green*, white matter skeleton; *red-yellow*, obese greater than non-obese group (*p* < 0.05, corrected); *blue-light blue*, obese lower than non-obese group (*p* < 0.05, corrected).

**FIGURE 2:**
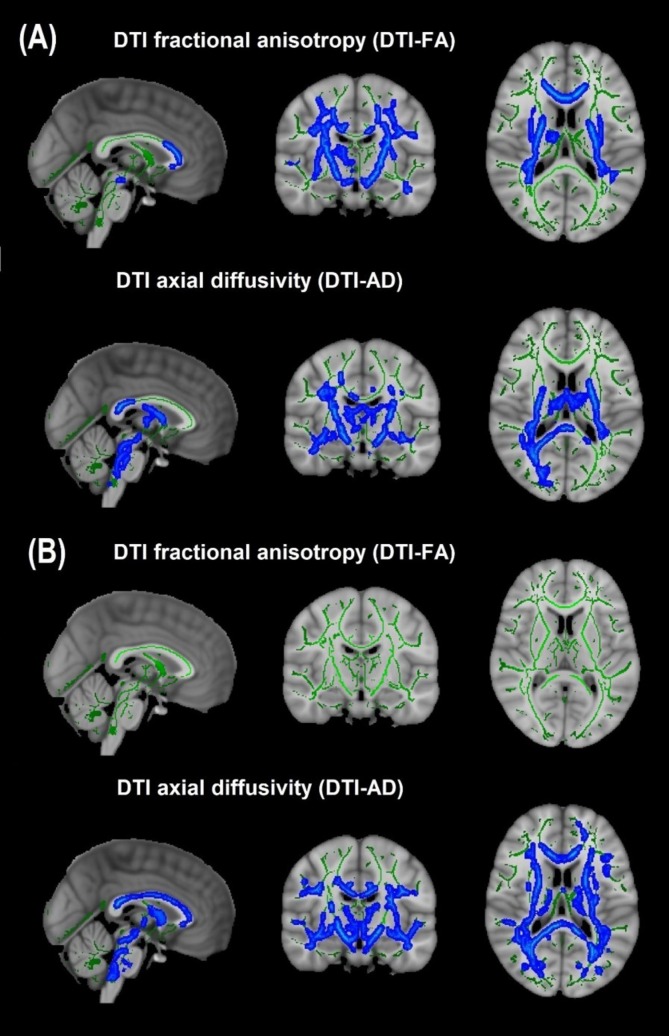
Diffusion tensor imaging-derived measures of white matter integrity in Cohort 1 and Cohort 2. **(A)** Cohort 1: Obese individuals had lower white matter fractional anisotropy (FA) and axial diffusivity (AD) than non-obese individuals. **(B)** Cohort 2: Obese individuals had similar white matter FA but lower AD than non-obese individuals. *Green*, white matter skeleton; *blue-light blue*, obese lower than non-obese group (*p* < 0.05, corrected).

Cohort 1: In TBSS analyses that covaried age, sex, race, and motion parameters, DBSI-FA was lower in obese compared to non-obese individuals, while DBSI-AD and RD were greater in obese compared to non-obese individuals (Figures 1A–C). DBSI-FF was lower in obese compared to non-obese individuals (Figure 1D) while DBSI-RF and DBSI-HF were greater in obese compared to non-obese individuals (Figures 1E,F). Lower DTI-FA and DTI-AD were observed in obese compared to non-obese individuals (Figure 2A). DTI-MD and DTI-RD were not significantly different between obese and non-obese individuals (data not shown).

Cohort 2: In TBSS analyses that covaried age, sex, race, and motion parameters, similar to Cohort 1, the obese group had lower DBSI-FF and greater DBSI-RF when compared to the non-obese group (Figures 1D,E). DBSI-FA and DBSI-AD were greater in the obese compared to the non-obese group while DBSI-RD was lower in the obese group compared to the non-obese group (Figures 1A–C). DBSI-HF did not differ between obese and non-obese groups. DTI-AD was lower in the obese compared to the non-obese group (Figure 2B). DTI-FA (Figure 2B), DTI-MD and DTI-RD were not significantly different between obese and non-obese individuals (data not shown).

**FIGURE 3:**
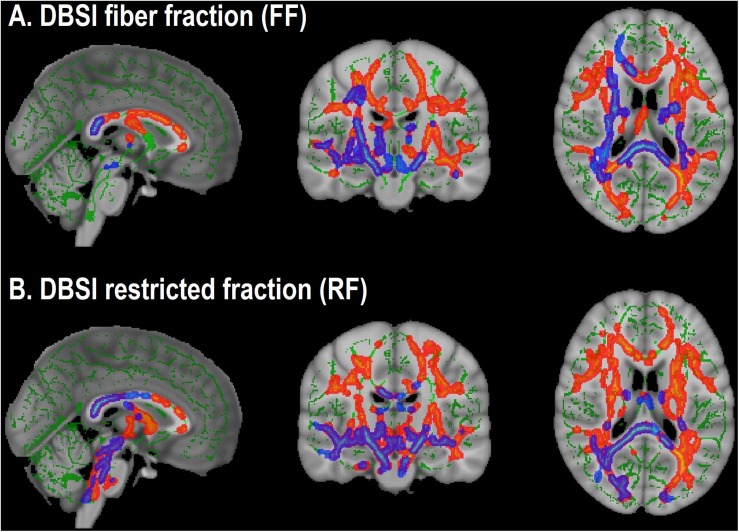
Overlap in white matter tracts with significant differences between obese and non-obese groups in both cohorts (Cohort 1: *Blue-purple*; Cohort 2: *Red-yellow*). **(A)** Lower DBSI fiber fraction in obese compared to non-obese. **(B)** Greater DBSI restricted fraction in obese compared to non-obese.

For the group differences in DBSI-FF and DBSI-RF, we determined the degree to which WM tracts overlapped in both cohorts (Figure 3). Differences in DBSI-RF and DBSI-FF were observed in widespread WM tracts and the percentage of overlap between both cohorts in all 48 WM tracts are included in Supplementary Table 1, in which columns are sorted in descending order according to the number of voxels that overlap for DBSI-RF.

### Voxelwise Correlations Between BMI and DBSI Metrics of Interest

Higher BMI related to greater voxelwise DBSI-RF in WM tracts within obese and non-obese groups in Cohort 1 but not within either group in Cohort 2. BMI did not relate to voxelwise DBSI-FF in WM tracts in either group in either cohort (data not shown).

### ROI Analyses of Hippocampal and Amygdalar DBSI Metrics

**FIGURE 4:**
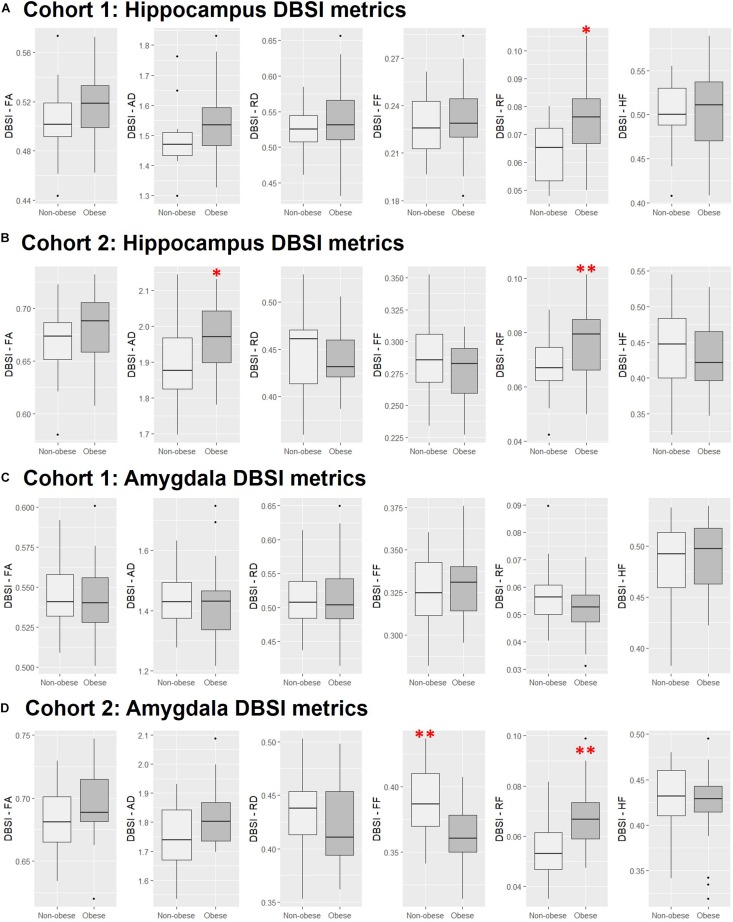
Comparison of DBSI metrics in the hippocampus **(A,B)** and amygdala **(C,D)** between obese and non-obese groups in Cohort 1 and Cohort 2. FA, fractional anisotropy; AD, axial diffusivity; RD, radial diffusivity; FF, fiber fraction; HF, hindered fraction. Median, first and third quartiles, 1.5 × interquartile range shown. ^∗^, ^∗∗^, *p* ≤ 0.05, 0.01, respectively.

In both Cohort 1 and Cohort 2, we compared average hippocampal and amygdalar volumes and hippocampal and amygdalar DBSI-derived metrics (DBSI-FA, DBSI-AD, DBSI-RD, DBSI-FF, DBSI-RF, DBSI-HF) between obese and non-obese groups using multiple linear regression, covarying for age, sex, race, average hippocampal or amygdalar volumes, and motion measures (volume-by-volume translation and rotation) (Figure 4 and Tables 2, 3). In Cohort 1, hippocampal DBSI-RF was greater in the obese group when compared to the non-obese group (Cohen’s *d* effect size = 1.03; 19.7% increase). Amygdalar DBSI metrics were not different between obese and non-obese groups in Cohort 1. In Cohort 2, hippocampal DBSI-AD and DBSI-RF were greater in the obese compared to the non-obese group (Cohen’s *d* effect sizes = 0.59 and 0.70, 3.4% increase and 12.2% increase, respectively) and amygdalar DBSI-FF and DBSI-RF were lower and greater in the obese compared to the non-obese group, respectively (Cohen’s *d* effect sizes = 1.2 for both comparisons, 8.3% decrease and 22% increase, respectively). Amygdalar volume was larger in obese relative to non-obese individuals in both cohorts. Hippocampal volumes and other DBSI-derived metrics were not different between obese and non-obese individuals in either cohort.

**TABLE 2:** Hippocampal DBSI metrics and volumes in obese and non-obese groups in Cohort 1 and Cohort 2.

	**Cohort 1**			**Cohort 2**		
	**Obese**	**Non-obese**	***p*-value**	**Obese**	**Non-obese**	***p*-value**
DBSI-FA	0.52 ± 0.03	0.5 ± 0.03	0.06	0.68 ± 0.03	0.67 ± 0.03	0.37
DBSI-AD	1.54 ± 0.11	1.48 ± 0.09	0.09	1.97 ± 0.1	1.9 ± 0.1	0.02^∗^
DBSI-RD	0.54 ± 0.05	0.53 ± 0.03	0.35	0.44 ± 0.03	0.45 ± 0.04	0.67
DBSI-FF	0.23 ± 0.02	0.23 ± 0.02	0.37	0.28 ± 0.02	0.29 ± 0.03	0.22
DBSI-RF	0.08 ± 0.01	0.06 ± 0.01	0.045^∗^	0.08 ± 0.01	0.07 ± 0.01	0.008^∗^
DBSI-HF	0.51 ± 0.05	0.50 ± 0.04	0.35	0.43 ± 0.05	0.44 ± 0.06	0.54
Hippocampal volume (mm^3^)	4203.2 ± 308	4009.2 ± 274	0.89	4358.8 ± 810	3856.1 ± 356	0.67

*Mean ± S.D. shown. DBSI metrics are unitless. **^∗^**, **^∗∗^**, ^∗∗∗^, *p* ≤ 0.05, 0.01, 0.001 relative to non-obese. DBSI, Diffusion Basis Spectrum Imaging; FA, fractional anisotropy; AD, axial diffusivity; RD, radial diffusivity; FF, fiber fraction; RF, restricted fraction; HF, hindered fraction.*

**TABLE 3:** Amygdalar DBSI metrics and volumes in obese and non-obese groups in Cohort 1 and Cohort 2.

	**Cohort 1**			**Cohort 2**		
	**Obese**	**Non-obese**	***p*-value**	**Obese**	**Non-obese**	***p*-value**
DBSI-FA	0.54 ± 0.02	0.55 ± 0.02	0.3	0.7 ± 0.03	0.68 ± 0.02	0.41
DBSI-AD	1.43 ± 0.12	1.44 ± 0.09	0.61	1.83 ± 0.11	1.75 ± 0.11	0.31
DBSI-RD	0.51 ± 0.06	0.51 ± 0.04	0.26	0.42 ± 0.04	0.43 ± 0.03	0.16
DBSI-FF	0.33 ± 0.02	0.33 ± 0.02	0.27	0.36 ± 0.02	0.39 ± 0.03	<0.001^∗∗∗^
DBSI-RF	0.05 ± 0.01	0.06 ± 0.01	0.33	0.07 ± 0.01	0.05 ± 0.01	<0.001^∗∗∗^
DBSI-HF	0.49 ± 0.04	0.49 ± 0.04	0.28	0.42 ± 0.05	0.43 ± 0.03	0.78
Amygdala volume (mm^3^)	1746.04 ± 157	1669.41 ± 116	0.07	1802.5 ± 338	1575.7 ± 117	0.05^∗^

*Mean ± S.D. shown. DBSI metrics are unitless. ^∗^, ^∗∗^, ^∗∗∗^, *p* ≤ 0.05, 0.01, 0.001 relative to non-obese. DBSI, diffusion basis spectrum imaging; FA, fractional anisotropy; AD, axial diffusivity; RD, radial diffusivity; FF, fiber fraction; RF, restricted fraction; HF, hindered fraction.*

### Cognitive Correlations With Hippocampal and Amygdalar DBSI-RF and DBSI-FF

**FIGURE 5:**
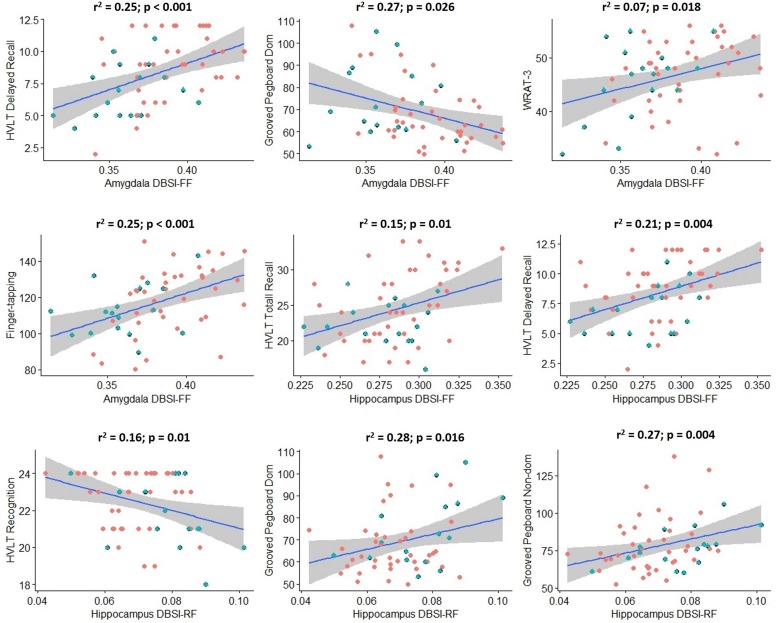
Significant correlations between hippocampus/amygdala DBSI fiber fraction and DBSI restricted fraction with performance in key measures of cognition in Cohort 2 (blue: obese; red: non-obese). Shaded area surrounding regression lines are 95% confidence intervals). DBSI-FF, fiber fraction; DBSI-RF, restricted fraction; HVLT, Hopkins Verbal Learning Test; WRAT-3, Wide Range Achievement Test 3; Dom, dominant; Non-dom, Non-dominant.

Scores from cognitive measures acquired from Cohort 2 were correlated across obese and non-obese individuals with DBSI-RF and DBSI FF in the hippocampus and amygdala, controlling for age (Figure 5). Eighteen cognitive measures had enough data points to be included in these exploratory analyses. BVMT, WAIS-III digit span subtest and FTT for the non-dominant hand performances were not included due to insufficient data points (≥20 subjects did not have one or more of these data points). All other correlations between ROI DBSI-FF or DBSI-RF and cognitive measures were not significant (*p* ≥ 0.06; data not shown). Also, we compared cognitive measure scores to assess between-group differences (data not shown). The obese group showed lower total recall (HVLT total recall, *p* = 0.02) and lower delayed verbal recall (HVLT delayed recall; *p* = 0.007), while no differences were observed in other cognitive measures (*p* ≥ 0.11).

## Discussion

The findings of our current study indicate the presence of diffuse neuroinflammation (greater DBSI-RF) in several WM tracts and hippocampus in both cohorts and amygdala in Cohort 2 and lower apparent axonal density (DBSI-FF) in several WM tracts in both cohorts and amygdala in Cohort 2 in obese individuals as assessed by DBSI. Additionally, obese groups had consistently higher DBSI-AD when compared to non-obese groups, but DBSI-FA and DBSI-RD were inconsistent across cohorts (lower DBSI-FA and greater DBSI-RD in Cohort 1; greater DBSI-FA and lower DBSI-RD in Cohort 2). These findings might also indicate that neuroinflammation-related processes (cellular infiltration and tissue edema) could have confounded DTI-derived metrics. Exploratory analyses showed correlations between hippocampal and amygdalar DBSI-RF or DBSI-FF and some cognitive variables in Cohort 2.

### Comparison Between DTI and DBSI Findings in Obesity

Diffusion tensor imaging has been extensively used to evaluate WM microstructure changes associated with obesity. Consistently, lower DTI-FA has been observed in obese compared to non-obese groups (Kullmann et al., 2015), while DTI-AD and DTI-RD alterations have been mixed (Mueller et al., 2011; Xu et al., 2013; Kullmann et al., 2016; Mazza et al., 2017; Papageorgiou et al., 2017). In our study, in several WM tracts, DTI-AD was lower in obese groups in both cohorts, DTI-FA was lower in the obese group in Cohort 1, while DTI-RD was not different between groups in either cohort. Using the traditional interpretation of DTI results, these findings indicate impaired overall WM integrity and axonal injury in the obese groups. Because DTI models both intra-axonal and extra-axonal water diffusion, cellularity associated with obesity might lead to decreased diffusion in all directions, resulting in the observed decrease in DTI-AD even without the presence of axonal injury. When isotropic diffusion was accounted for in the DBSI modeling, DBSI-AD was slightly greater in the obese groups in both cohorts, which could indicate increased water diffusion parallel to the axons in the extracellular compartment as a result of increased tissue edema. The presence of tissue edema could also contribute to the lower apparent axonal density (lower DBSI-FF) in the obese groups. These findings indicate that neuroinflammation-related processes (cellular infiltration and tissue edema) could have confounded DTI-derived metrics. This notion was previously suggested by some authors and demonstrated by histopathological studies in animal models of neuroinflammatory diseases (Wang et al., 2014; Cross and Song, 2017; Winklewski et al., 2018; Zhan et al., 2018).

The inconsistent differences in DBSI-FA and DBSI-RD (lower DBSI-FA and greater DBSI-RD in Cohort 1; greater DBSI-FA and lower DBSI-RD in Cohort 2) could also support the hypothesis that different biological processes may underlie obesity-related WM microstructure alterations (Haley et al., 2018). In each cohort, the pattern of change in DBSI-FA, DBSI-AD, and DBSI-RD might represent a different stage of WM reorganization post-injury. This hypothesis has been used to explain the bi-directional changes in DTI-FA in other conditions (e.g., a rodent model of traumatic brain injury) (Harris et al., 2016). In the case of obesity, the underlying mechanism could be an ongoing process of WM structural reorganization (demyelination/remyelination, loss of long WM tracts, axon sprouting) associated with persistent neuroinflammatory process. Nevertheless, although this hypothesis is a plausible explanation for the inconsistent diffusivity differences observed in our study, these differences might also be due to between-cohort differences in hardware used, such as head coils, and DTI acquisition parameters or participant characteristics due to sample selection criteria. As mentioned above, while DTI data from Cohort 2 participants were primarily selected from a convenience sample comprising the HIV- control group of an HIV neuroimaging study, Cohort 1 participants were selected specifically for a study of brain alterations in obesity unconfounded by current or past co-morbid disease. Therefore, more stringent screening for diabetes, mental illness, substance and alcohol abuse, and IQ/education was performed in Cohort 1 relative to Cohort 2. We excluded individuals with diabetes in order to study neuroinflammation in obesity *per se*, unconfounded by hyperglycemia and insulin resistance, factors already linked to neuroinflammation (Pugazhenthi et al., 2017). More rigorous experimental designs are necessary to confirm the validity of these hypotheses and to determine what non-BMI factors relate to DBSI measures.

### Histopathological Abnormalities Associated With Neuroinflammation in Obesity

In obese groups, greater DBSI-RF in WM tracts and hippocampus presumably reflect an increase in CNS resident inflammatory cells. Significant increases in glial fibrillary acidic protein (GFAP) immunoreactive astrocytes were observed in the hippocampus and frontal and parietal cortices in rodent models of obesity (Tomassoni et al., 2013). Evidence of increased gliosis was observed in the mediobasal hypothalamus of living obese humans assessed by MRI (Thaler et al., 2012; Schur et al., 2015), which related to greater post-mortem GFAP staining intensity (Schur et al., 2015). Peripheral inflammation as indicated by plasma fibrinogen related to alterations in DTI-measured apparent diffusivity in the amygdala (Cazettes et al., 2011). In several rodent studies, obesity-induced microglia activation and other types of neuroinflammation were frequently observed in hypothalamus, hippocampus, amygdala, and other brain regions (Erion et al., 2014; Tucsek et al., 2014; Guillemot-Legris and Muccioli, 2017). Obesity-related microglial activation in rodents mediates the relationship between synaptic dysfunction and cognitive deficits, which are blocked by inhibition of microglial activation (Erion et al., 2014; Cope et al., 2018). Reversal of obesity-related macrophage infiltration into leaky BBB improves obesity-associated cognitive dysfunction (Stranahan et al., 2016). Since DBSI doesn’t distinguish different cell types, it would be necessary to perform validation studies, e.g., correlation of DBSI-derived metrics with histopathological measures of neuroinflammation in rodent models of obesity and in human postmortem brain, to confirm that these altered properties truly reflect neuroinflammation and detect which cell types are responsible for the observed changes in DBSI-RF in our study.

Interestingly, in Cohort 1, DBSI-HF was greater in obese compared to non-obese individuals. DBSI-HF models non-restricted water diffusion in the extracellular compartment and reflects tissue edema in acute neuroinflammatory conditions (Wang et al., 2011; Wang et al., 2014; Zhan et al., 2018). In the case of possible chronic neuroinflammation in disease states such as obesity, histopathological evaluation is needed to determine the mechanism that underlies greater DBSI-HF.

### Neuroinflammation, Axonal Density, and Cognitive Performance

In the current study, we showed that DBSI-RF, an indicator of neuroinflammation, and DBSI-FF, an indicator of axonal density, in hippocampus and amygdala related to cognitive performance in some measures. These results are in line with studies in which impaired performance on memory tasks is induced by hippocampal neuroinflammation in rodents with diet-induced obesity (Pistell et al., 2010; Beilharz et al., 2016; Cope et al., 2018) and the observed association between plasma fibrinogen and water diffusion in the amygdala in obese and overweight individuals (Cazettes et al., 2011). The current results suggest altered water diffusivity in the brain, and perhaps neuroinflammation, may relate to altered cognitive performance. However, these data should be considered preliminary since analyses were exploratory and not corrected for multiple comparisons. Future studies may determine whether putative neuroinflammation modulates the relationship between BMI and cognitive performance.

### Limitations and Future Directions

The primary strength of the current study is the replication of the findings that DBSI-RF, a putative marker of neuroinflammation, and DBSI-FF, a marker of axonal density, are greater and lower, respectively, in obese than non-obese individuals in two independent cohorts. The main weakness of this study is that data were not available to link DBSI metrics to alterations in inflammation-related behavior or proinflammatory cytokines in plasma or CSF. Without histopathological validation, though plausible, it remains speculative that the DBSI-measured alterations truly reflect neuroinflammation. Previous studies showed that DBSI-RF is associated with activated microglia and astrogliosis in several neuroinflammatory conditions (Wang et al., 2011; Chiang et al., 2014; Wang et al., 2014) but this has not been examined in obesity. Interestingly, the regions of increased DBSI-RF and decreased DBSI-FF in obese individuals in Cohort 1, from a study designed to test for differences in the brain due to obesity unconfounded by other health issues, falls almost entirely within the regions of the findings from obese individuals in Cohort 2, a convenience sample, as described above. Lack of convergent findings for some DBSI anisotropic metrics could be due to variations in stage of WM reorganization and differences between cohorts including participant characteristics and DTI sequence parameters, as discussed above. A third weakness is that age, sex and race distributions differed between obese and non-obese individuals in one or both cohorts. There are age, sex, and racial differences in adiposity and associated traits including systemic inflammation severity due to physiological, social and psychological factors (Thorand et al., 2006; Stepanikova et al., 2017a, b). While we controlled for age, sex and race in our data analyses, we cannot rule out the possibility that differences between groups in these factors contributed to our results. Clearly, age, sex, and race should be included in future studies as variables of primary interest with sufficient sample size to power these studies. DTI sequence parameters were slightly different between cohorts, which prevented us from combining data across cohorts. Future studies should be prospective in nature, include larger sample sizes and obtain complimentary measures of neuroinflammation using PET with radiotracers specific for activated microglia, plasma and CSF inflammatory marker levels, and measures of cognitive function. Also, studies of animal models of obesity would allow for histopathological validation of DBSI metrics.

## Conclusion

In two independent cohorts, we showed that a DBSI-derived indicator of neuroinflammation is greater and axonal density is lower in obese compared to non-obese humans. In addition, the discrepancies between DBSI- and DTI-derived anisotropic metrics demonstrate the limitations of DTI when applied to disease states that may be accompanied by neuroinflammation. Additionally, these findings highlight the significance of applying multi-component models of diffusion imaging in these populations. Future studies are warranted to determine whether high-calorie diet-induced neuroinflammation occurs in ROIs outside hippocampus, amygdala, and hypothalamus and its potential role in obesity-associated impairment in behaviors thought to be regulated by these regions. Finally, the results of the current study indicate that putative neuroinflammation and associated cognitive impairment occurs even in obese individuals without diabetes. Given the evidence implicating diabetes in the development of neuroinflammation and cognitive impairment (Pugazhenthi et al., 2017), it will be important to assess relationships between metabolic markers, cognition, and MRI-derived neuroinflammation metrics in individuals who do and do not develop insulin resistance over time. Also, further histopathological studies in postmortem brain are necessary to confirm that the altered DBSI properties we observed in obese humans truly reflect neuroinflammatory processes.

## Data Availability Statement

The datasets generated and used to perform analyses for this study are available upon a request to the corresponding author.

## Ethics Statement

All studies were approved by the Washington University School of Medicine Human Research Protection Office and were carried out in accordance with the principles expressed in the Declaration of Helsinki.

## Author Contributions

TH, NS, and SE contributed to the conception of the study. NS, JS, PS, JSS, BA, S-KS, TH, and SE provided the imaging datasets and the expertise in neuroimaging. AS, TM, JR, ON, NS, and SE conducted pre-processing, neuroimaging, and statistical analysis. AS, TM, and SE wrote the initial draft of the manuscript. All authors critically revised the manuscript, contributed to the interpretation of the results, and approved the final version.

## Conflict of Interest

The authors declare that the research was conducted in the absence of any commercial or financial relationships that could be construed as a potential conflict of interest.

## Footnotes

1 https://surfer.nmr.mgh.harvard.edu/

## Abbreviations

AD: axial diffusivity
BBB: blood-brain barrier
BMI: body mass index
CNS: central nervous system
CSF: cerebrospinal fluid
DBSI: Diffusion basis spectrum imaging
DTI: diffusion tensor imaging
FA: fractional anisotropy
FF: fiber fraction
GLM: general linear model
HF: hindered fraction
ICV: intra-cranial volume
MRI: magnetic resonance imaging
MS: multiple sclerosis
PET: positron emission tomography
RD: radial diffusivity
RF: restricted fraction
ROI: region of interest analysis
TBSS: tract-based spatial statistics
TFCE: Threshold-Free Cluster Enhancement
TSPO: translocator protein
WM: white matter
